# Substantially Enhanced Properties of 2D WS_2_ by High Concentration of Erbium Doping against Tungsten Vacancy Formation

**DOI:** 10.34133/2022/9840970

**Published:** 2022-07-04

**Authors:** Hongquan Zhao, Guoxing Zhang, Bing Yan, Bo Ning, Chunxiang Wang, Yang Zhao, Xuan Shi

**Affiliations:** ^1^Chongqing Institute of Green and Intelligent Technology, Chinese Academy of Sciences, China; ^2^University of Chinese Academy of Sciences, Beijing 100064, China; ^3^Chongqing University of Posts and Telecommunications, Chongqing 400065, China

## Abstract

Doping in 2D materials is an important method for tuning of band structures. For this purpose, it is important to develop controllable doping techniques. Here, we demonstrate a substitutional doping strategy by erbium (Er) ions in the synthesis of monolayer WS_2_ by chemical vapor deposition. Substantial enhancements in photoluminescent and photoresponsive properties are achieved, which indicate a tungsten vacancy suppression mechanism by Er filling. Er ion doping in the monolayer WS_2_ is proved by X-ray diffraction (XRD) and X-ray photoelectron spectra (XPS), fluorescence, absorption, excitation, and Raman spectra. 11.5 at% of the maximum Er concentration is examined by energy dispersive X-ray spectroscopy (EDX). Over 6 times enhancement of intensities with 7.9 nm redshift in peaks are observed from the fluorescent spectra of Er-doped WS_2_ monolayers compared with their counterparts of the pristine WS_2_ monolayers, which agrees well with the density functional theory calculations. In addition, over 11 times of dark current, 469 times of photocurrents, photoresponsivity, and external quantum efficiency, and two orders of photoresponse speed are demonstrated from the Er-doped WS_2_ photodetector compared with those of the pristine WS_2_ device. Our findings prove rare-earth doping in 2D materials, the exciting and ideal technique for substantially enhanced photoluminescent and photoresponsive properties.

## 1. Introduction

The study of photoluminescent and photoresponsive properties in two-dimensional (2D) semiconductors has attracted remarkable scientific and technological interests [1–5]. Among which, transition metal dichalcogenides (TMDs) with over 40 types of metal and chalcogen combinations is one of the mostly focused family due to their thickness-dependent band gaps from visible to near-infrared [6, 7], theoretically high switching ratios [8, 9], carrier mobilities [10, 11], and photoresponsivities for applications in photoelectric and electric fields [12–16]. As the first representative TMDs, 2D MoS_2_ is the mostly characterized material. Besides MoS_2_, WS_2_ is possibly more attractive due to its 20 times higher of photoluminescent efficiency, better thermal stability, larger valence band splitting, and theoretically reduced effective carrier masses [17–19]. However, a huge gap exists between the theoretical predictions and the experimental performances [20]. For instance, the measured mobilities and photoresponse time of monolayer WS_2_-based field-effect transistors (FETs) are typically in the range of 1-50 cm2/V∙s and 5-2000 ms, respectively [21–25], which are far less than the predicted mobility of >500 cm2/V∙s and photoresponse time of <100 ns, respectively [17, 26–29]. The reduced performances are partly attributed to the unoptimized device fabrications and structural designs [30]. The essential reason may due to the defect-induced trap states that produced in the synthesizing process [31], since electronic states at energy band edges and defects in 2D WS_2_ are significantly more active and especially fatal to its photoluminescent and photoresponsive performances compared with their bulk counterparts. 2D WS_2_ prepared by top-down approaches usually have better crystalline quality but with uncontrollable thicknesses and small sizes [32]. For practical applications, wafer-scale synthesis of homogeneous monolayer WS2 is required. In this regard, chemical vapor deposition (CVD) is extensively employed [22, 23, 33, 34]. Nevertheless, from the perspective of the second law of thermodynamics, a variety of defects are inevitably generated during CVD synthesis. The typical type of intrinsic defects comes from the missing atoms, including transition metal vacancies and chalcogen vacancies, which has been observed in many kinds of 2D TMDs [35–38]. The photoluminescence quantum yields of CVD-grown TMDs are only 0.01–6% of the mechanically exfoliated samples due to the high density of vacancy defects [39]. As the most defective CVD grown 2D TMDs, WSe_2_ shows 1.48 at% of Se vacancy, but the density of transition metal vacancies in TMDs and the corresponding influences on their photoelectric performances are still unknown [40]. Many methods, including high-temperature treatment, chemical reduction, and ultraviolet irradiation, have been proposed to reduce the chalcogen defects, while approach to lower the transition metal defects is still absent [41, 42]. Hence, it is striking to make clear of the impact caused by transition metal defects and find a solution to effectively reduce the metal defects in 2D semiconductors.

Chemical doping is usually employed to tune the functionality of semiconductors through doping-type and band structure modulations [43–46]. Rare-earth (RE) ions are known to have complicated energy levels due to their unique intra-4f electronic transitions [47], which is commonly doped in traditional insulator or semiconductor hosts to provide abundant excited energy levels, and enable the extension of their absorption and excitation spectrum width, enhancement of their quantum yields, photostabilities, and Stokes shifts [48, 49]. Recently, substitutional doping of TMDCs via CVD has been reported, and ferromagnetism characteristics has achieved [50, 51]. However, RE in situ doping strategies in 2D materials by CVD method are still in the early stage. The mechanism and impact of the RE substitution on the structural, electronic, and photonic properties of 2D monolayers are far from being answered [52].

Herein, a substitutional Er ion doping strategy in synthesis of large scale of 2D WS_2_ by CVD is proposed. By adding excessive Er_2_O_3_ into the tungstic acid (H_2_WO_4_) as the tungsten source, erbium ions are successfully in situ doped into the WS_2_ monolayer with a high concentration of 11.5 at% examined by energy dispersive X-ray spectroscopy (EDX). The high doping concentration may relate to the high vacancy density of tungsten in WS_2_ synthesized by CVD, and similar ion radii between Er and W ions in the WS_2_ matrix, which make it convenient for substitutional doping. Strong characteristic peaks contributed from erbium ions are observed both in the X-ray diffraction spectra, X-ray photoelectron spectra, and Raman spectra. The photoluminescent and photoresponsive performances are comprehensively and substantially enhanced by erbium doping compared with the counterparts of the pristine WS_2_ monolayers fabricated by the same method. Over 6 times of fluorescent intensities are observed in Er-doped spectra from 20 samples. Eleven excitation peaks from 761 nm to 1011 nm are found from the excitation spectra of Er-doped WS_2_ monolayers; those are nonexistent in the pristine WS_2_ monolayers. The fluorescence and absorption enhancement are well agreed with the calculation results of density functional theory (DFT). In addition, the Er-doped WS_2_ photodetectors show over 11 times of dark current, 469 times of photocurrent, photoresponsivity, and external quantum efficiency, and two orders of photoresponse speed compared with those of the pristine WS_2_ photodetectors fabricated and measured under the same course of processing. The combined multiple characterizations and theoretical calculations indicate that extra lattice distortion is not introduced by erbium doping, but the density of tungsten vacancy is effectively reduced leading to a reduced density of surface trapping states. In the meantime, significant higher density of carriers is provided by the introduction of substitutional erbium doping in the monolayer WS_2_ matrix.

## 2. Methods

### 2.1. Synthesis of WS_2_ via CVD Process

**Figure 1:**
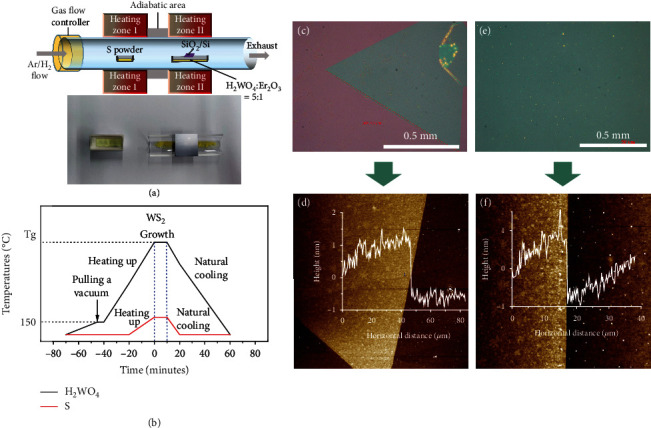
WS_2_(Er) and WS_2_ membranes fabricated by chemical vapor deposition (CVD) method. (a) Schematic illustration of the experimental setup for synthesis of WS_2_ and WS_2_(Er) films. (b) Temperature curve and gas flow control in the double temperature zones. (c) Typically synthesized triangular WS_2_(Er) membrane with the side length over 1 mm. (d) Thickness measured by AFM of the sample in (c). (e) Centimeter scale of WS_2_(Er) film with indefinite morphology. (f) Thickness measured by AFM of the sample in (e).

The preparation of 2D WS_2_ is carried out in quartz tubes with 2-inch diameter in double temperature furnaces, as is schematically shown in Figure 1(a). 3 × 3 cm^2^ of SiO_2_/Si wafers with 300 nm thickness of SiO_2_ is used as the substrates. 150 mg sulfur powder (>99.5%) is weighed as the precursor, tungstic acid (H_2_WO_4_) powder (>99.5%) with the dosage of 65 mg is used as the tungsten source, and 4-8 mg of sodium chloride (NaCl) powder (>99.99%) is used as the growth promoter. 13 mg of Er_2_O_3_ powder (>99.99%) is used as the RE dopant for the preparation of Er-doped WS_2_ membranes (WS_2_(Er)). The H_2_WO_4_, Er_2_O_3_, and NaCl powders are mixed sufficiently before being placed in the quartz boats. The SiO_2_/Si substrates are put directly face down above the quartz boat with a distance of 10 mm between the substrate and the powder.

In the beginning, the CVD chamber is vacuumed (<5 Pa), and argon gas (UHP, >99.999%) is fed subsequently at a rate of 300 sccm to reach a standard atmospheric pressure in the quartz tube. The temperature curve of the synthesis process is shown in Figure 1(b). In the meantime, the second temperature zone is heated to 150°C at a rate of 5°C/min, and then held for 5 min. The CVD chamber is vacuumed again during the heating process (<5 Pa), and then, the argon gas is fed at a rate of 300 sccm to reach a standard atmospheric pressure in the tube. The purpose of this step is to remove the impurity gases and desorbed dusts generated by heating of the quartz chamber. Subsequently, the first and second temperature zones are heated to 200°C and 850°C (Tg) in 40 minutes, respectively. The synthesis of WS_2_ is finished in 10 min in an argon-hydrogen mixture gas (UHP, Ar 90%, H_2_ 10%) flow at a rate of 60 sccm. Finally, the samples are naturally cooled to room temperature in a 60 sccm of argon flow.

### 2.2. Characterization of the Pristine and Er-Doped WS_2_ Membranes

The surface morphologies of the pristine and Er-doped WS_2_ samples are characterized by optical microscope (50i POL, Nikon) and energy dispersive spectrum (EDS) in SEM (JSM-7800F, JEOL), and the roughness and thickness of WS_2_ samples are characterized by atomic force microscope (AFM, Dimension EDGE, Bruker). The Raman spectra of WS_2_ and WS_2_(Er) samples are measured by a laser confocal Raman spectrometer (InVia Reflex, Renishaw) with a continue wave (cw) laser wavelength of 532 nm. X-ray diffraction spectra (X'Pert3 Powder) of the samples are performed to confirm the diffracted characteristic peaks of the doped erbium ions. Photoluminescence (PL) spectra are measured by a laser scanning confocal microscope system excited at 532 nm. A spherical aberration correction TEM (Titan Cubed Themis G2 300) is used for the measurement of HAADF-STEM and energy dispersive X-ray spectroscopy (EDX) to characterize the crystalline structures and elemental compositions of the WS_2_(Er) samples.

## 3. Results and Discussion

By slightly adjusting the weight of NaCl, up to 1 mm of triangular shape and even centimeter-scale of Er-doped WS_2_ membranes can be synthesized by the CVD method. Typical optical micrographs of the WS_2_ samples are shown in Figures 1(c) and 1(e), and their corresponding thicknesses are measured to be nearly 1 nm by AFM, which is consistent with the reported thickness of monolayer WS_2_, as shown in Figures 1(d) and 1(f) [53, 54]. The pristine WS_2_ are synthesized under the same conditions without Er_2_O_3_ as a dopant. Relevant experimental details can be found in S1 (Supporting Information).

**Figure 2:**
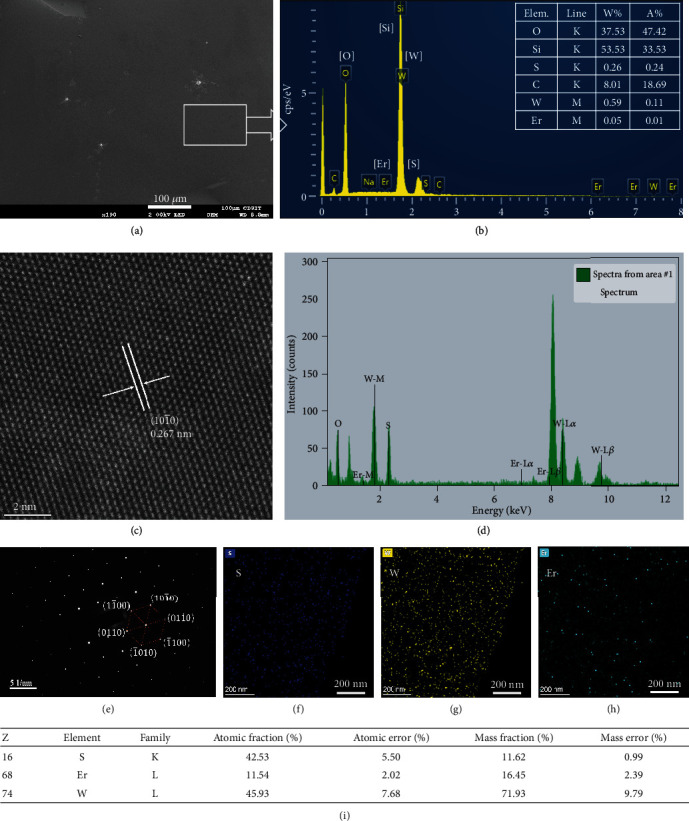
Microscopic analysis of WS_2_(Er) monolayer by SEM and TEM. (a) SEM and (b) EDS image of a WS_2_(Er) membrane synthesized on a SiO_2_/Si substrate. The inserted table in (b) shows the atomic ratio measured by EDS in a selected rectangular region in (a). (c) WS_2_(Er) membrane on Cu grid characterized by HAADF-STEM with the correction of spherical aberration. (d) EDX image of the WS_2_(Er) membrane measured in (c). (e) SAED pattern of the same WS_2_(Er) membrane. (f), (g), and (h) are the elemental mapping image of [S], [W], and [Er] in the WS_2_(Er) membrane measured by the HAADF-STEM system, respectively. (i) Atomic fraction of [S], [W], and [Er] elements in the WS_2_(Er) matrix analyzed by the HAADF-STEM system.

Compared with surface chemical doping approaches, substitutional doping involves covalent bonding and is a more stable doping technique. Caption substitutional doping in TMDs, including niobium, rhenium, erbium, and holmium, has been demonstrated to tune their electronic properties in recent report [45, 52, 54, 55]. However, the doping concentrations are usually low (typically ≦1 at%), and the PL intensities even decreased. To get the maximum Er doping concentration, excessive dosage of Er_2_O_3_ is added into the tungsten source with a weight ratio of 1 : 5 to the H_2_WO_4_ powder, but the exact concentration of Er ion in the WS_2_ film is still unknown. SEM and EDS are firstly used to evaluate the [Er] concentration.  Figure 2(a) and Figure 2(b) show the SEM image and selected area elemental analysis by EDS of the Er-doped WS_2_ film. The insert chart in Figure 2(b) shows the elemental type, weight, and atomic ratio of the WS_2_ film on SiO_2_/Si substrates measured by EDS in SEM. Besides high contents of [O] and [Si] from SiO_2_ substrates, there are still tiny amount of [S], [W], and [Er] which are examined from the EDS spectrum. Compared with the atomic ratio of [W], the atomic percentage of [Er] to [W] is close to 10%. The high content of [C] may come from the remnant of organic cleaning. To further confirm the concentration of erbium ions in the WS_2_ film, the WS_2_(Er) sample is characterized by high-angle annular darkfield scanning transmission electron microscopy (HAADF-STEM) with the correction of spherical aberration, as shown in Figure 2(c). A crystalline plane spacing of 0.267 nm can be measured between two immediate lines along the (10$\overset{¯}{1}$0) direction, which is consistent with the results of monolayer WS_2_ [22]. Because the atomic number of [Er] (No. 68) is close to but a little smaller than [W] (No. 74), and their ion radius in WS_2_ crystalline is similar, it is difficult to distinguish [Er] locations from the HAADF-STEM image of WS_2_ film by brightness or ion size. But for the same reason, it is also reasonable to infer that minimal crystalline distortions are induced in the WS_2_ monolayer by Er doping. Substitutional doping of caption ions with large differences in ion radius usually induces large lattice distortions, which in principle leads to additional surface defects. Surface trapping states caused by these defects will further degenerate material properties, including quantum yields, carrier mobilities, photoresponse speed, and especially fatal for 2D materials.  Figure 2(d) shows the EDX image of the WS_2_(Er) sample on TEM Cu grid; a weak Er-M peak implies the existence of doped [Er] atoms with the Er atomic ratio of about 11.5 at% in WS_2_ analyzed by the system, as shown in Figure 2(i). This atomic ratio is close to the result of about 10 at% measured by the EDS-SEM for the sample on SiO_2_/Si substrate. A strong [O] peak is also found in the EDX image, which may come from the residue of PMMA or organic contaminations from organic cleaning of the substrates, since PMMA is used to transfer the WS_2_(Er) membrane onto the TEM Cu grid. The selective area electron diffraction (SAED) diagram of the Er-doped WS_2_ film shows six diffraction points on the diffraction pattern, which corresponds to tungsten and sulfur sublattices, respectively, indicating the intact hexagonal symmetry of the present WS_2_ film after high concentration of Er doping (Figure 2(e)). Additionally, elemental mapping images (Figures 2(f)–2(h)) show the homogeneous distribution of [S], [W], and [Er] in the sample, which also suggests that Er atoms are successfully doped into the WS_2_ matrix.

**Figure 3:**
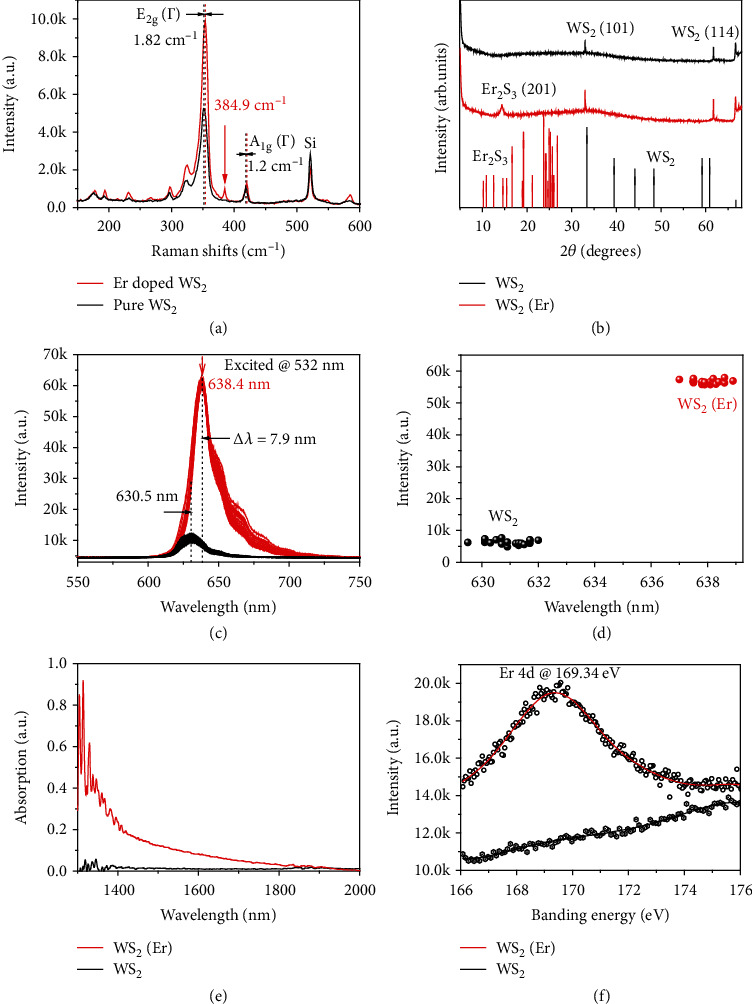
Comparison of different spectra between WS_2_ and WS_2_(Er) monolayer. (a) Raman spectra, (b) X-ray diffraction spectra, and (c) PL spectra of the CVD synthesized WS_2_ and WS_2_(Er) membrane measured in the same conditions for comparison. 20 PL spectra of WS_2_ and WS_2_(Er) triangles on SiO_2_/Si substrates are measured under the same conditions and shown in (c). (d) Distribution of the intensity and wavelength of the PL peak in (c) for statistical analysis. (e) Absorption spectra of WS_2_ and WS_2_(Er) membranes measured from 1300 nm to 2000 nm. (d) High-resolution XPS spectra of Er 4d for WS_2_(Er) and pure WS_2_ membranes for comparison.

Subsequently, Raman, X-ray diffraction, X-ray photoelectron, photoluminescence, and absorption spectra of the prepared WS_2_(Er) samples are performed, and the results are compared with the counterparts of the pristine WS_2_ samples.  Figure 3(a) presents Raman spectra of the prepared WS_2_ and WS_2_(Er) films under 532 nm laser excitation. The WS_2_(Er) sample shows almost equal peak intensity in the out-of-plane vibration mode (A_1g_*(Г)*), but two times intensity of the in-plane vibration mode (E_2g_*(Г)*) compared with that of the pristine WS_2_ sample. Besides, 1.82 cm^−1^ and 1.2 cm^−1^ of redshifts are observed in the E_2g_*(Г)* and A_1g_*(Г)* from the WS_2_(Er) sample, respectively. Both of these phenomena indicate the possible effects of Er doping in WS_2_ matrix. Because intensity of the A_1g_*(Г)* mode strongly depends on the layer thickness, the nearly equal intensities of the A_1g_*(Г)* mode indicate little thickness difference between these two samples. On this basis, higher defect density usually leads to the lower crystalline symmetry and thus to the lower of E_2g_*(Г)* intensity. Two times intensity enhancement of the E_2g_*(Г)* mode possibly indicates the lower defect concentration in the WS_2_(Er) sample. In addition, a Raman peak located at 384.9 cm^−1^ is obviously seen from the WS_2_(Er) sample but nonexistent in the WS_2_ sample. This peak is very likely contributed from the Tg mode of Er ion [56].

X-ray diffraction spectra are measured on WS_2_ and WS_2_(Er) films to qualitatively confirm the successful doping of Er ions. Because Bragg diffraction effect is weak on a monolayer sample, thick films with large sizes are required in this measurement. In the meantime, concentration of Er ions in the WS_2_ matrix is not dependent on the thickness and size of the films. Thus, WS_2_ and WS_2_(Er) samples with about 10 nanometer in thicknesses and several millimeter in sizes are used in this measurement.  Figure 3(b) shows the X-ray diffraction spectra of the WS_2_ and WS_2_(Er) samples. The WS_2_ (101) and WS_2_ (104) diffraction peaks are observed in both of the two type of samples. Meanwhile, a strong extra peak is discovered in the WS_2_(Er) sample located at the position of 2*θ* = 14.4°, which is highly consistent with the X-ray diffraction peak of Er_2_S_3_ (201). The strength ratio between this peak and WS_2_(101) peaks is 0.51, further indicating the high concentration of Er doping in the WS_2_(Er) sample.

**Figure 4:**
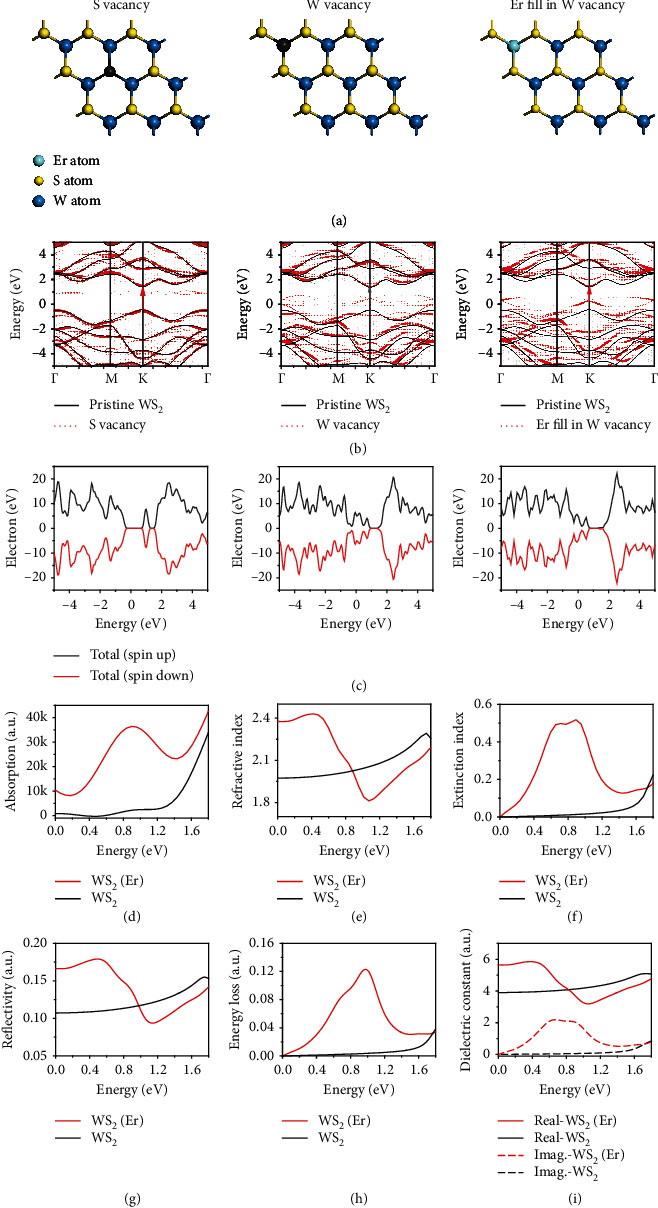
First principle calculations on the S-vacancy, W-vacancy, and W-vacancy filled by Er of a 3 × 3 × 1 WS_2_ supercell. (a) Schematic illustration of the S-vacancy, W-vacancy, and W-vacancy filled by Er of WS_2_ matrix. (b) Energy band structures corresponding to the S-vacancy, W-vacancy, and Er-doped conditions of WS_2_ supercell described in (a). Weighted bubble chart is used and displayed by the red dotted lines representing the band structures of vacancy and Er-doped WS_2_ monolayers, while the black solid curves represent band structure of the protocell WS_2_. (c) PDOS of monolayer WS_2_ under the S-vacancy, W-vacancy, and Er filled in W-vacancy conditions, respectively. (d)–(i) Dependences of absorption index, refractivity index, extinction index, reflectivity, energy loss, and dielectric constants, respectively, on the incident photon energy (from 0 to 1.8 eV) of the pristine WS_2_ and WS_2_(Er) monolayer.

PL spectra of the two types of monolayers are performed under 532 nm laser excitation. Interestingly, over 6 times enhancement of the PL intensity and 7.9 nm redshift of the peak wavelength are achieved in the WS_2_(Er) monolayer. No obvious broadening is observed by comparing FWHM of the PL peaks from WS_2_(Er) and WS_2_ monolayers, indicating that very slight energy transfer occurs after doping. To better identify the spectral change, the same experiments are carried out on 20 WS_2_ and WS_2_(Er) triangles, respectively, and similar results are obtained as shown in Figure 3(c). Their statistical variations of the PL intensities and peak positions are shown in Figure 3(d). The small variations in the intensities and peak positions of the same type of samples may due to the fluctuation of laser power densities and slight inhomogeneous thicknesses. However, the significantly enhanced PL intensities and redshifts of the peak positions between the WS_2_ and WS_2_(Er) samples should originate from the Er doping. The possible reasons may lie in three factors: Firstly, the formation of tungsten vacancy in the CVD synthesis is probably suppressed by the doped Er ions and thus the suppression of nonradiative recombinations. It is well known that tungsten transition metal sulfides usually have much higher of vacancy density compared with that of the molybdenum transition metal sulfides. The quantum efficiency increases substantially as the tungsten vacancy is effectively reduced by the Er ion filling. Secondly, additional defects caused from crystalline distortion by the introduction of Er ions into WS_2_ matrix is negligible, which requires similar ion radii between Er and W in the WS_2_ crystalline. Thirdly, the energy bands become more rich and active by the introduction of Er ions, since complex and rich electronic levels exist in Er ions. The 7.9 nm of photoluminescent redshift between WS_2_ and WS_2_(Er) samples implies the occurrence of shallow impurity level transitions in the PL process.  Figure 3(e) shows the absorption spectra from 1300 nm to 2000 nm of the WS_2_(Er) and pure WS_2_ films on SiO_2_/Si substrates measured by an infrared spectrum imager (Spotlight, Platinum Elmer). It is obvious that the WS_2_(Er) film shows peak absorption coefficiency at 1314 nm and with much higher of absorption coefficency compared with that of the WS_2_ film from 1300 nm to 1800 nm. This result is highly consistent with the calculated absorption characteristics based on DFT as shown in Figure 4(d). Furthermore, X-ray photoelectron spectra (Thermo scientific K-Alpha+) are performed on both of the two films. A strong Er 4d peak is seen to be located at 169.34 eV as shown in Figure 3(f), which is assigned to Er^3+^ [57].

To further confirm our deduction, the first principle calculations based on the density functional theory (DFT) are performed on the S-vacancy, W-vacancy, and W-vacancy filled by Er of WS_2_ monolayers, respectively. Electronic band structures and partial density of states (PDOS) of these WS_2_ monolayers are calculated by generalized gradient approximation (GGA) method. Because 11.5 at% of Er is measured in the EDX spectra, 3 × 3 × 1 supercell is used in the calculation corresponding to 11 at% of [Er] concentration to [W] atoms. Band unfolded technique and weighted bubble chart are applied for the vacancy and Er-filled band structures. The calculation details are described in the supporting information S2.  Figure 4(a) shows the schematic images of the S-vacancy, W-vacancy, and Er filled in W-vacancy of monolayer WS_2_, respectively.  Figure 4(b) shows the energy band structures corresponding to Figure 4(a). The weighted red bubble charts in Figure 4(b) represent the energy bands of the corresponding vacancy and Er-filled WS_2_ monolayers, while the black curves represent the energy bands of the WS_2_ protocell for comparison. For S-vacancy WS_2_ monolayer, the energy bands change slightly compared with the protocell WS_2_, except two deep defect energy levels with low weight in the forbidden band. High weight of valance band maximum (VBM) and conduction band minimum (CBM) exist in the high symmetric *K* point in Brillouin zone, which indicates a relatively small impact of the S-vacancy on the direct transition. For W-vacancy WS_2_ monolayer, the energy bands change to more complicated. Multiple valance levels extend to the center of the forbidden band with uniform of low weight, and the VBM shows very small weight in the*K*point; thus, a very low possibility of carrier exists in the*K*point, implying that a very low possibility of direct transition occurs in the*K*point. Furthermore, when W-vacancy is filled by Er ion, the VBM moves upward in the *K* point with much higher of weight, indicating much higher of possibility for direct transition, and thus the enhancement of the PL efficiency with a redshift. In comparison of these three conditions, S-vacancy in the WS_2_ monolayer leads to relatively small impact on the PL efficiency and minute peak shift. W-vacancy degenerates the PL efficiency more seriously than S-vacancy condition. When Er filled in W-vacancy, the PL intensity is enhanced greatly together with a redshift in the PL peak, which well interprets the experimental results as mentioned above.  Figure 4(c) shows the PDOS of the S-vacancy, W-vacancy, and Er-filled WS_2_ monolayer. Figures 4(d)–4(i) show the dependences of indexes, including refractivity, extinction, reflectivity, energy loss, and dielectric constants, respectively, on the incident photon energy of the pristine WS_2_ and WS_2_(Er) monolayer. Notably, the WS_2_(Er) sample shows much higher of absorption, extinction, energy loss, and imaginary part of dielectric indexes in the infrared region centered at 0.85 eV, while its refractivity, reflectivity, and real part of dielectric indexes show amplitude exchanges in the infrared region centered at 0.85 eV compared with the pristine WS_2_ monolayer. To understand the effects of Er doping with lower concentrations, 4 × 4 × 1 and 5 × 5 × 1 supercells of the S-vacancy, W-vacancy, and Er filled in W-vacancy of WS_2_ monolayers are also calculated and discussed in detail in supporting information S2.

**Figure 5:**
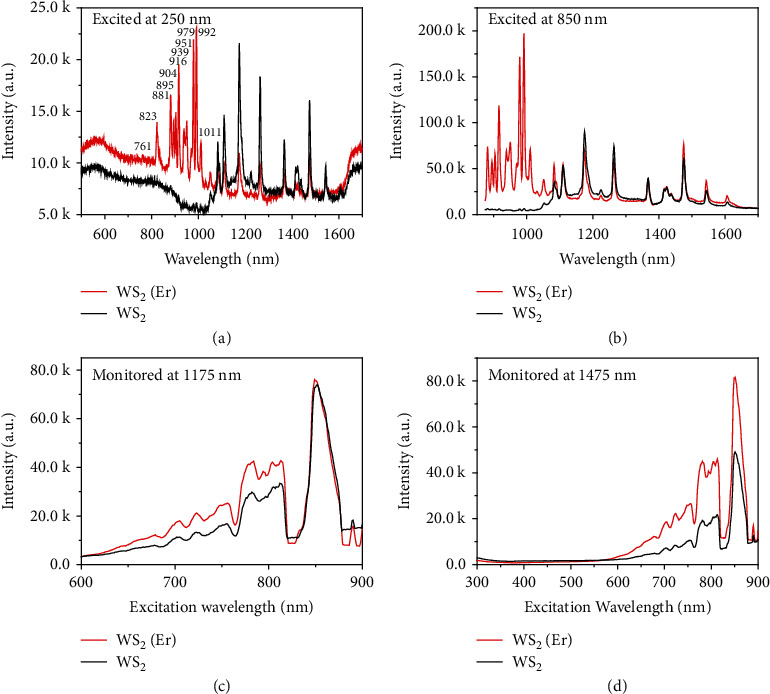
Comparison of the excitation spectra between WS_2_ and WS_2_(Er) monolayer. The excitation spectrum is measured at 77 K under the excitation wavelength of (a) 250 nm and (b) 850 nm. Excitation spectra by scanning of the excitation wavelength are also measured and the photoluminescent intensities are monitored at (c) 1175 nm and (d) 1475 nm, respectively.

To understand the effect of Er doping in photoluminescence under different excitations from ultraviolet to infrared, the excitation spectra excited under 250 nm and 850 nm on the WS_2_ and WS_2_(Er) samples are performed at 77 K, as shown in Figures 5(a) and 5(b), respectively. When the samples are excited at 250 nm, the WS_2_(Er) sample exhibits higher fluorescence intensity from 500 nm to 1100 nm. In particular, 11 strong and sharp PL peaks are apparently observed from 761 nm to 1011 nm in the WS_2_(Er) sample, but nonexistent in the WS_2_ sample. The appearance of these peaks is undoubtedly due to the impurity levels in the forbidden band introduced by the doped Er ions, especially the Er intra-4f levels, which has strong localization characteristics, stable optical excitation, and recombination process. The 11 sharp peaks are probably originated from the transitions from ^4^F_9/2_ to the ground state of ^4^I_15/2_ (736 nm), from ^4^I_9/2_ to ^4^I_15/2_ (804 nm), and from ^4^I_11/2_ to ^4^I_15/2_ (980 nm), respectively.^55^ Because of the orbital interactions between the Er-4f and W-4d orbits, the original energy levels split into multiple sublevels, which leads to the present 11 sharp spectra terms at 77 K. This result proves the rich optical characteristics and new features of 2D materials by Er doping. When the excitation wavelength changes to 850 nm, similar result is obtained, and 9 sharp peaks from 881 nm to 1011 nm are observed (Figure 5(b)). The excitation spectra of the WS_2_ and WS_2_(Er) monolayers by scanning the excitation wavelength and under the monitor wavelength of 1175 nm and 1475 nm for photoluminescence are shown in Figures 5(c) and 5(d), respectively. When the excitation wavelength scans from 600 nm to 900 nm and the photoluminescent intensity is monitored at 1175 nm, both the WS_2_ and WS_2_(Er) samples show increasing fluorescent efficiency from 600 to 815 nm, but the WS_2_(Er) sample shows a higher fluorescent efficiency compared with WS_2_ sample. The maximum peak intensities are seen when excited at 849 nm for both of the samples. Similarly, when the excitation wavelength is scanned from 300 nm to 900 nm, and the PL intensity is monitored at 1475 nm, the PL intensities increase in the excitation region from 600 nm to 815 nm for both of the samples, and the WS_2_(Er) sample shows over two times of efficiency compared with the counterpart of WS_2_ sample. The maximum peak intensity of the two samples also appears at the excitation wavelength of 849 nm. Combined with the PL measurements by a 532 nm laser excitation, the photoluminescent results prove the enhancement of quantum efficiency from ultraviolet to infrared region of the Er-doped WS_2_.

**Figure 6:**
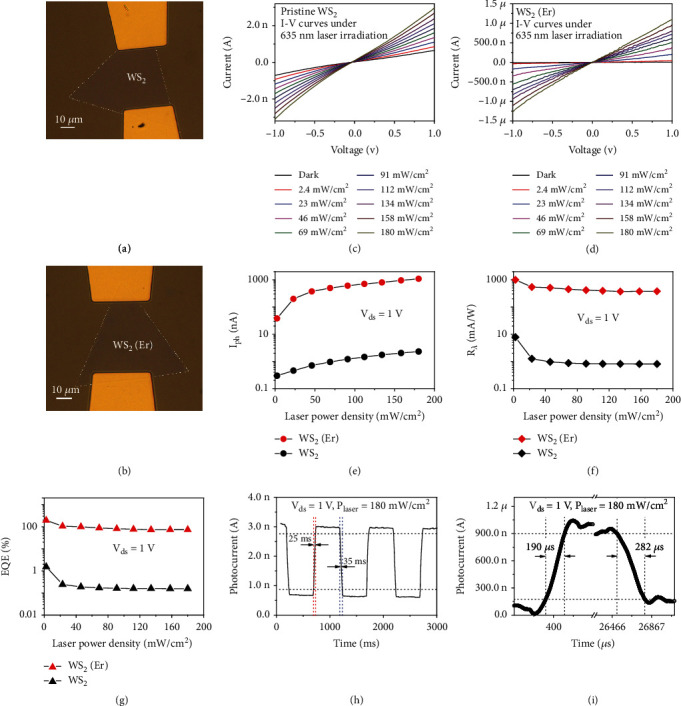
Photoresponsive characteristics of the WS_2_ and WS_2_(Er) devices. (a) and (b) are optical micrographs of the photodetectors based on WS_2_ and WS_2_(Er) monolayers, respectively. *I-V* and photocurrents of the (c) pristine WS_2_ and (d) WS_2_(Er) photodetectors under a 635 nm cw laser illumination at different laser power densities are demonstrated. Photocurrents of both samples in the condition of *V*_ds_ = 1 V and illuminated at different power densities are extracted from (c) and (d) and shown in (e). (f) Photoresponsivities and (g) external quantum efficiencies of both samples are evaluated based on (e). Photoresponse time of the (h) pristine WS_2_ and (i) WS_2_(Er) photodetectors under the condition of *V*_ds_ = 1 V and 180 mW/cm^2^ of illumination for on states are also demonstrated. Lines to connect the points in (e), (f), and (g) are guides for eyes.

Researches concerning the doping effects of WS_2_ monolayers on the photoresponse characteristics are of interests, and photodetectors based on monolayer WS_2_ and WS_2_(Er) with similar thickness, morphologies, and sizes are fabricated under the same conditions with Au electrodes for ohmic contacts. Triangular side length of the WS_2_ and WS_2_(Er) samples are 73 *μ*m and 71 *μ*m, respectively. The length of the channels between two electrodes of these two types of devices is both 40 *μ*m. Their optical micrographs are shown in Figures 6(a) and 6(b). *I–V* and photoresponse characteristics of the monolayer devices are measured on a shielded probe station connected to a Keithley semiconductor analyzer (SCS4200) by a standard DC technique. Details of the *I–V* curves in dark condition of the WS_2_ and WS_2_(Er) devices are discussed in the supporting information (S3). The *I–V* curves of the WS_2_ and WS_2_(Er) devices illuminated by a 635 nm diode laser at different incident intensities from dark to 180 mW/cm^2^ are shown in Figures 6(c) and 6(d), respectively. Compared with the pristine WS_2_ monolayer device, the WS_2_(Er) device shows 11 times of dark currents at *V*_ds_ = 1 V (S3), and 469 times of photocurrents at *V*_ds_ = 1 V, and under 180 mW/cm^2^ of illumination. Photocurrent values of both samples under different illumination power densities are extracted and shown in Figure 6(e) for comparison. Moreover, critical parameters including photoresponsivity (*R*_*λ*_) and external quantum efficiency (EQE) are evaluated by the following equations [58, 59]:

(1) $$\begin{array}{c} R_{\lambda }=\left(I_{\text{photo}}-I_{\text{dark}}\right)/\left(P\cdot S\right), \end{array}$$

(2) $$\begin{array}{c} \text{EQE}=\left(h\cdot c\cdot R_{\lambda }\right)/\left(2\pi \cdot e\cdot \lambda \right), \end{array}$$

where *I*_photo_ and *I*_*dark*_ are photocurrent and dark current, respectively; *P* is the illumination laser power density; *SS* is the effective area under illumination; *e* is the electronic charge; *h* is the Planck constant; and *c* is the light velocity. Based on the experimental data in Figure 6(e), *R*_*λ*_ and EQE at different illumination power densities from 2.4 to 180 mW/cm^2^ are calculated for WS_2_ and WS_2_(Er) devices, and shown in Figures 6(f) and 6(g), respectively. The WS_2_ and WS_2_(Er) devices show the maximum *R*_*λ*_ of 7.8 mA/W and 994.5 mA/W at the *V*_ds_ = 1 V and 2.4 mW/cm^2^ of illumination, respectively. Similarly, the WS_2_ and WS_2_(Er) devices show the maximum EQE of 1.5% and 194.6% at the *V*_ds_ = 1 V and 2.4 mW/cm^2^ of illumination, respectively. According to Equations (1) and (2), the*R*_*λ*_andEQEonly depend on the photocurrents; as a result, the maximum ratio of*R*_*λ*_andEQEbetween the two type of devices is also 469 times at*V*_ds_ = 1 Vand 180 mW/cm^2^ of illumination. This result provides a powerful proof that the doped Er ion contributes to the photoelectric efficiency in a large scale due to its active and rich electronic energy levels.

WS_2_(Er) monolayer is proved to be photosensitive, and its potential in fast photodetector applications is also demonstrated from the photoswitching characteristics, as shown in Figures 6(h) and 6(i). A high-performance graphic sampling multimeter (Keithley DMM7510) accompanied with a chopper-modulated 635 nm cw laser and a DC voltage supplier is used to measure the photoswitching behavior of the WS_2_ and WS_2_(Er) devices at *V*_ds_ = 1 V and 180 mW/cm^2^ of illumination for on states. 25 ms of rising time and 35 ms of falling time are measured from the WS_2_ device. In comparison, the WS_2_(Er) device shows over two orders enhancement of the photoresponse speed with the rising time of 190 *μ*s (1/132 of 25 ms) and falling time of 282 *μ*s (1/124 of 35 ms) from one period of illumination. Notably, this speed is also over 200 times faster than the monolayer MoS_2_ device, and 20 times faster than the WS_2_ device with 6 nm thickness of WS_2_ [60, 61]. More comparisons are provided in the supporting information S4. The photoresponse speed is strongly depended on the density of surface trapping states, since surface states lead to the parasitic capacitance. Two orders enhancements of the photoresponse speed indicate the drastic decrease of the density of surface states in monolayer WS_2_(Er). Although multitype of sources including intrinsic defects, impurities, surface dangling bonds, and oxidations will lead to surface states, the main cause is still the intrinsic vacancy defects due to the inert surface feature of 2D materials. Therefore, the present two orders enhancement in the photoresponse speed provides further evidence for the vacancy filling mechanism by Er against the formation of tungsten vacancies in the CVD synthesis process.

## 4. Conclusion

In summary, high concentration of substitutional Er doping strategy in the synthesis of large scale of 2D WS_2_ by CVD is achieved experimentally. 11.5 at% of Er concentration in the WS_2_(Er) monolayer is examined by EDX. The high doping concentration may relate to the nature of high tungsten vacancy density formed during the CVD synthesis process. In addition, strong characteristic peaks contributed from the erbium ions are observed both in the X-ray diffraction spectra, X-ray photoelectron spectra, and the Raman spectra. The photoluminescent, absorptive, and photoresponsive performances are comprehensively and substantially enhanced by the erbium doping compared with the counterparts of the pristine WS_2_ monolayers synthesized by the same method. Over 6 times of fluorescent intensities are observed from the WS_2_(Er) spectra. The fluorescent enhancements and redshifts are well agreed with the calculation results by density functional theory (DFT). Eleven excitation peaks from 761 nm to 1011 nm are found from the excitation spectra of WS_2_(Er) monolayer, and those are nonexistent in the pristine WS_2_ monolayer. In addition, the WS_2_(Er) photodetectors show over 11 times of dark current, 469 times of photocurrent, photoresponsivity, and external quantum efficiency, and over two orders of photoresponse speed compared with those of the pristine WS_2_ photodetectors fabricated and measured under the same conditions. The combined characterizations and theoretical calculations indicate that extra lattice distortions are not obviously introduced by the substitutional erbium doping, but the defect density is effectively reduced, which leads to a reduced density of surface trapping states, and the significantly higher density of carriers are provided by the introduction of substitutional Er ions in the monolayer WS_2_ matrix.

## Data Availability

All data needed to evaluate the conclusions in the paper are present in the paper and/or the Supplementary Materials. Additional data related to this paper may be requested from the authors.

## Supplementary Materials

Supplementary Materials S1 Details of the CVD synthesis for large scale of WS2 membranes by Er doping S2. First principle calculations S2.1. 4 × 4 supercell for S-vacancy, W-vacancy, and Er filled in W-vacancy conditions S2.2. 5 × 5 supercell for S-vacancy, W-vacancy, and Er filled in W-vacancy conditions S3. Dark current of photodetectors based on WS2 and WS2(Er) monolayers S4. Comparison of the photoresponsive characteristics between WS2(Er) photodetector and photodetector based on other 2D materials

## References

[1] Gong Y., Lin J., Wang X. (2014). Vertical and in-plane heterostructures from WS_2_/MoS_2_ monolayers. *Nature Materials*.

[2] Huang C., Wu S., Sanchez A. M. (2014). Lateral heterojunctions within monolayer MoSe_2_-WSe_2_ semiconductors. *Nature Materials*.

[3] Li D., Chen M., Sun Z. (2017). Two-dimensional non-volatile programmable p-n junctions. *Nature Nanotech*.

[4] Sahoo P. K., Memaran S., Xin Y., Balicas L., Gutierrez H. R. (2018). One-pot growth of two-dimensional lateral heterostructures via sequential edge-epitaxy. *Nature*.

[5] Zhao H., Mao Y., Mao X. (2018). Band structure and photoelectric characterization of GeSe monolayers. *Advanced Functional Materials*.

[6] Sayan R., Bermel P. (2018). Electronic and optical properties of ultra-thin 2D tungsten disulfide for photovoltaic applications. *Solar Energy Materials and Solar Cells*.

[7] Yorulmaz B., Özden A., Sar H., Ay F., Sevik C., Perkgöz N. K. (2019). CVD growth of monolayer WS_2_ through controlled seed formation and vapor density. *Materials Science in Semiconductor Processing*.

[8] Zhang S., Dong N., McEvoy N. (2015). Direct observation of degenerate two-photon absorption and its saturation in WS_2_ and MoS_2_ monolayer and few-layer films. *ACS Nano*.

[9] Wang Q. H., Kalantar-Zadeh K., Kis A., Coleman J. N., Strano M. S. (2012). Electronics and optoelectronics of two-dimensional transition metal dichalcogenides. *Nature Nanotechnology*.

[10] Zhang Y., Zhang Y., Ji Q. (2013). Controlled growth of high-quality monolayer WS_2_ layers on sapphire and imaging its grain boundary. *ACS Nano*.

[11] Zheliuk O., Lu J., Yang J., Ye J. (2017). Monolayer superconductivity in WS_2_. *Physica Status Solidi (RRL) – Rapid Research Letters*.

[12] Yao J., Zheng Z., Yang G. (2016). Layered-material WS_2_ topological insulator Bi_2_Te_3_ heterostructure photodetector with ultrahigh responsivity in the range from 370 to 1550 nm. *Journal of Materials Chemistry C*.

[13] Yin W., Liu X., Zhang X. (2020). Synthesis of tungsten disulfide and molybdenum disulfide quantum dots and their applications. *Chemistry of Materials*.

[14] Choi W., Choudhary N., Han G. H., Park J., Akinwande D., Lee Y. H. (2017). Recent development of two-dimensional transition metal dichalcogenides and their applications. *Materials Today*.

[15] Wang X.-R., Shi Y., Zhang R. (2013). Field-effect transistors based on two-dimensional materials for logic applications. *Chinese Physics B*.

[16] Lee H. S., Lee D., Hwang S. W., Hwang E., Jena D., Yoo J. W. (2014). High-performance photocurrent generation from two-dimensional WS2field-effect transistors. *Applied Physics Letters*.

[17] Liu L., Kumar S. B., Ouyang Y., Guo J. (2011). Performance limits of monolayer transition metal dichalcogenide transistors. *IEEE Transactions on Electron Devices*.

[18] Zhu Z. Y., Cheng Y. C., Schwingenschlögl U. (2011). Giant spin-orbit-induced spin splitting in two-dimensional transition-metal dichalcogenide semiconductors. *Physical Review B*.

[19] Zhao W., Ghorannevis Z., Chu L. (2013). Evolution of electronic structure in atomically thin sheets of WS_2_ and WSe_2_. *ACS Nano*.

[20] Jariwala D., Sangwan V. K., Lauhon L. J., Marks T. J., Hersam M. C. (2014). Emerging device applications for semiconducting two-dimensional transition metal dichalcogenides. *ACS Nano*.

[21] Reale F., Palczynski P., Amit I. (2017). High-mobility and high-optical quality atomically thin WS_2_. *Scientific Reports*.

[22] Lan C., Li C., Yin Y., Liu Y. (2015). Large-area synthesis of monolayer WS_2_ and its ambient-sensitive photo-detecting performance. *Nanoscale*.

[23] Lan C., Zhou Z., Zhou Z. (2018). Wafer-scale synthesis of monolayer WS_2_ for high-performance flexible photodetectors by enhanced chemical vapor deposition. *Nano Research*.

[24] Wang Q., Zhang Q., Zhao X. (2019). High-energy gain upconversion in monolayer tungsten disulfide photodetectors. *Nano Letters*.

[25] Bersch B. M., Eichfeld S. M., Lin Y.-C. (2017). Selective-area growth and controlled substrate coupling of transition metal dichalcogenides. *2D Materials*.

[26] Kaasbjerg K., Thygesen K. S., Jacobsen K. W. (2012). Phonon-limited mobility in n-type single-layer MoS_2_ from first principles. *Physical Review B*.

[27] Kaasbjerg K., Thygesen K. S., Jauho A.-P. (2013). Acoustic phonon limited mobility in two-dimensional semiconductors: deformation potential and piezoelectric scattering in monolayer MoS_2_ from first principles. *Physical Review B*.

[28] Moody G., Tran K., Lu X. (2018). Microsecond valley lifetime of defect-bound excitons in monolayer WSe_2_. *Physical Review Letters*.

[29] Manzeli S., Ovchinnikov D., Pasquier D., Yazyev O. V., Kis A. (2017). 2D transition metal dichalcogenides. *Nature Reviews Materials*.

[30] Li X., Lin M. W., Basile L. (2016). Isoelectronic tungsten doping in monolayer MoSe_2_ for carrier type modulation. *Advanced Materials*.

[31] Ding K., Fu Q., Nan H., Gu X., Ostrikov K., Xiao S. (2021). Controllable synthesis of WS_2(1-x)_Se_2x_ monolayers with fast photoresponse by a facile chemical vapor deposition strategy. *Materials Science and Engineering: B*.

[32] Mak K. F., McGill K. L., Park J., McEuen P. L. (2014). The valley hall effect in MoS_2_ transistors. *Science*.

[33] Yu Y., Li C., Liu Y., Su L., Zhang Y., Cao L. (2013). Controlled Scalable Synthesis of Uniform, High-Quality Monolayer and Few- layer MoS_2_ Films. *Scientific Reports*.

[34] Gao Y., Liu Z., Sun D. M. (2015). Large-area synthesis of high-quality and uniform monolayer WS_2_ on reusable Au foils. *Nature Communications*.

[35] Zhou W., Zou X., Najmaei S. (2013). Intrinsic structural defects in monolayer molybdenum disulfide. *Nano Letters*.

[36] Nguyen L., Komsa H. P., Khestanova E. (2017). Atomic defects and doping of monolayer NbSe_2_. *ACS Nano*.

[37] Zhang S., Wang C. G., Li M. Y. (2017). Defect structure of localized excitons in a WSe_2_ monolayer. *Physical Review Letters*.

[38] He Y. M., Clark G., Schaibley J. R. (2015). Single quantum emitters in monolayer semiconductors. *Nature Nanotechnology*.

[39] Zheng B., Zheng W., Jiang Y. (2019). WO_3_-WS_2_ vertical bilayer heterostructures with high photoluminescence quantum yield. *Journal of the American Chemical Society*.

[40] Ding S., Lin F., Jin C. (2021). Quantify point defects in monolayer tungsten diselenide. *Nanotechnology*.

[41] Akinwande D., Brennan C. J., Bunch J. S. (2017). A review on mechanics and mechanical properties of 2D materials—graphene and beyond. *Extreme Mechanics Letters*.

[42] Li Y., Shi X., Dai F. (2020). Enhancement of photodetection by PbSe quantum dots on atomic-layered GeS devices. *Journal of Physics D: Applied Physics*.

[43] Zhang K., Feng S., Wang J. (2015). Manganese doping of monolayer MoS_2_: the substrate is critical. *Nano Letters*.

[44] Das S., Demarteau M., Roelofs A. (2015). Nb-doped single crystalline MoS2 field effect transistor. *Applied Physics Letters*.

[45] Bai G., Yuan S., Zhao Y. (2016). 2D layered materials of rare-earth Er-doped MoS_2_ with NIR-to-NIR down- and up-conversion photoluminescence. *Advanced Materials*.

[46] Lin Y. C., Dumcenco D. O., Komsa H. P. (2014). Properties of individual dopant atoms in single-layer MoS_2_: atomic structure, migration, and enhanced reactivity. *Advanced Materials*.

[47] Huang P., Zheng W., Zhou S. (2014). Lanthanide-doped LiLuF4 upconversion nanoprobes for the detection of disease biomarkers. *Angewandte Chemie International Edition*.

[48] Gai S., Li C., Yang P., Lin J. (2014). Recent progress in rare earth micro/nanocrystals: soft chemical synthesis, luminescent properties, and biomedical applications. *Chemical Reviews*.

[49] Binnemans K. (2009). Lanthanide-based luminescent hybrid materials. *Chemical Reviews*.

[50] Fu S., Kang K., Shayan K. (2020). Enabling room temperature ferromagnetism in monolayer MoS_2_ via in situ iron-doping. *Nature Communications*.

[51] Chen M., Hu C., Luo X., Hong A., Yu T., Yuan C. (2020). Ferromagnetic behaviors in monolayer MoS2 introduced by nitrogen-doping. *Physics Letters*.

[52] Zhang K., Bersch B. M., Joshi J. (2018). Tuning the electronic and photonic properties of monolayer MoS_2_ via in situ rhenium substitutional doping. *Advanced Functional Materials*.

[53] Shen J., Zhan L., Wang C. (2021). Isomeric compound dendrites on a monolayer WS_2_ substrate: morphological engineering and formation mechanism. *ACS Applied Nano Materials*.

[54] Chen X., Lin Z.-Z. (2018). A primary exploration to quasi-two-dimensional rare-earth ferromagnetic particles: holmium-doped MoS_2_ sheet as room-temperature magnetic semiconductor. *Journal of Nanoparticle Research*.

[55] Suh J., Park T. E., Lin D. Y. (2014). Doping against the native propensity of MoS_2_: degenerate hole doping by cation substitution. *Nano Letters*.

[56] Wang J. C., Zhu Y. Y. (2014). Study on the structural properties of polycrystalline Er_2_O_3_ films on Si (001) substrates by Raman spectra. *Advanced Materials Research*.

[57] Lun M., Wu W., Xing Z. (2020). Upconversion photoluminescence of Er^3+^ and Yb^3+^ codoped MoS_2_ powders. *Journal of Luminescence*.

[58] Xue D. J., Liu S. C., Dai C. M. (2017). GeSe thin-film solar cells fabricated by self-regulated rapid thermal sublimation. *Journal of the American Chemical Society*.

[59] Xin Y., Wang X., Chen Z. (2020). Polarization-sensitive self-powered type-II GeSe/MoS_2_ van der Waals heterojunction photodetector. *ACS Applied Materials & Interfaces*.

[60] Yin Z., Li H., Li H. (2012). Single-layer MoS_2_ phototransistors. *ACS Nano*.

[61] Perea-López N., Elías A. L., Berkdemir A. (2013). Photosensor device based on few-layered WS2 films. *Advanced Functional Materials*.

