# Supplementary material for: Substantially Enhanced Properties of 2D WS2 by High Concentration of Erbium Doping against Tungsten Vacancy Formation
Source: Research (Wash D C). 2022 Jul 4;2022:9840970. doi: 10.34133/2022/9840970 (PMC9285636; doi:10.34133/2022/9840970)
Supplement: Supplementary Materials — S1 Details of the CVD synthesis for large scale of WS2 membranes by Er doping S2. First principle calculations S2.1. 4 × 4 supercell for S-vacancy, W-vacancy, and Er filled in W-vacancy conditions S2.2. 5 × 5 supercell for S-vacancy, W-vacancy, and Er filled in W-vacancy conditions S3. Dark current of photodetectors based on WS2 and WS2(Er) monolayers S4. Comparison of the photoresponsive characteristics between WS2(Er) photodetector and photodetector based on other 2D materials [file 9840970.f1.docx]

**Supporting information**

Substantially Enhanced Properties of 2D WS_2_ by High Concentration of Erbium Doping against Tungsten Vacancy Formation

Hongquan Zhao^1^*, Guoxing Zhang^1,2^, Bing Yan^1,2^, Bo Ning^2,3^, Chunxiang Wang^1^, Yang Zhao^3^, Xuan Shi^1^*

**Content**

**S1. Details of the CVD synthesis for large scale of WS_2_ membranes by Er doping**

**S2. First principle calculations**

**S2.1. 4×4 supercell for S-vacancy, W-vacancy, and Er filled in W-vacancy conditions**

**S2.2. 5×5 supercell for S-vacancy, W-vacancy, and Er filled in W-vacancy conditions**

**S3. Dark current of photodetectors based on WS_2_ and WS_2_(Er) monolayers**

**S4. Comparison of the photoresponsive characteristics between WS_2_(Er) photodetector and photodetector based on other 2D materials**

**References**

**S1. Details of the CVD synthesis for large scale of WS_2_ membranes by Er doping**

Both pristine WS_2_ and Er doped WS_2_ are synthesized by CVD method. The CVD setups and the temperature setting for the double-heating furnace are schematically shown in **Figure 1a** and 1b. Sulfur powder is used as the precursor, and H_2_WO_4_ and NaCl powders are used as the tungsten source and the growth promoter, respectively. WS_2_ are synthesized on SiO_2_/Si (thickness of SiO_2_ = 300 nm) substrates in Ar/H_2_ (10% of H_2_ concentration) atmosphere.

When a small amount of NaCl is mixed into the H_2_WO_4_ powder, volatile transition metal halide oxides with high reactivity can be formed at a relatively low temperature. The addition of small amount of NaCl can also reduce the vulcanization energy barrier for growth of WS_2_, thus increase the growth rate along the in-plane direction.^[1-2]^ The mixed H_2_ in the Ar/H_2_ airflow plays a key role of reducing agent in the reaction process. The chemical reactions for the H_2_WO_4_, NaCl, H_2_, and S that take place during the synthetic process are as follows:

$H_{2}WO_{4}\to WO_{3}+H_{2}O$(1)

$$2WO_{3}+2NaCl\to WO_{2}{Cl}_{2}+Na_{2}{WO}_{4} (2)$$

$$WO_{2}{Cl}_{2}+4H_{2}+3S\to WS_{2}+2HCl+2H_{2}O+H_{2}S (3)$$

By modifying the dosage of H_2_WO_4_ and sulfur powder, and the synthesis temperature, different sizes of WS_2_ membranes with different morphologies have been synthesized by slightly adjust the dosages of the promoter NaCl. In this work, the dosage of H_2_WO_4_ and sulfur powder is 65 mg and 150 mg, respectively, and the synthesizing temperature is 850 ℃. When 2-4 mg dosage of NaCl is mixed into the H_2_WO_4_ powder, the synthesized WS_2_ membranes usually show triangular shape with the diameter ranging from one hundred to several hundreds micrometers as we shown in **Figure S1a** and Figure S1b. When the dosage of promoter NaCl increase to about 6 mg, the triangular WS_2_ membranes start to coalesce, and WS_2_ membranes with indefinite mophologies are appeared with the sizes ranging from one millimeter to several millimeter as shown in Figure S1c. Further slightly increase the dosage of NaCl to 8 mg will lead to the centimeter level of WS_2_ membranes with indefinite mophologies, but grain boundaries are usually seen on the surfaces, as we shown in Figure S1d. It is noted that when 1 : 5 (in weight to H_2_WO_4_ powder) or lower dosage of Er_2_O_3_ powder is added and mixed sufficiently into the tungsten powder, the morphology change of the synthesized WS_2_(Er) membrane is not observed in the experiments.


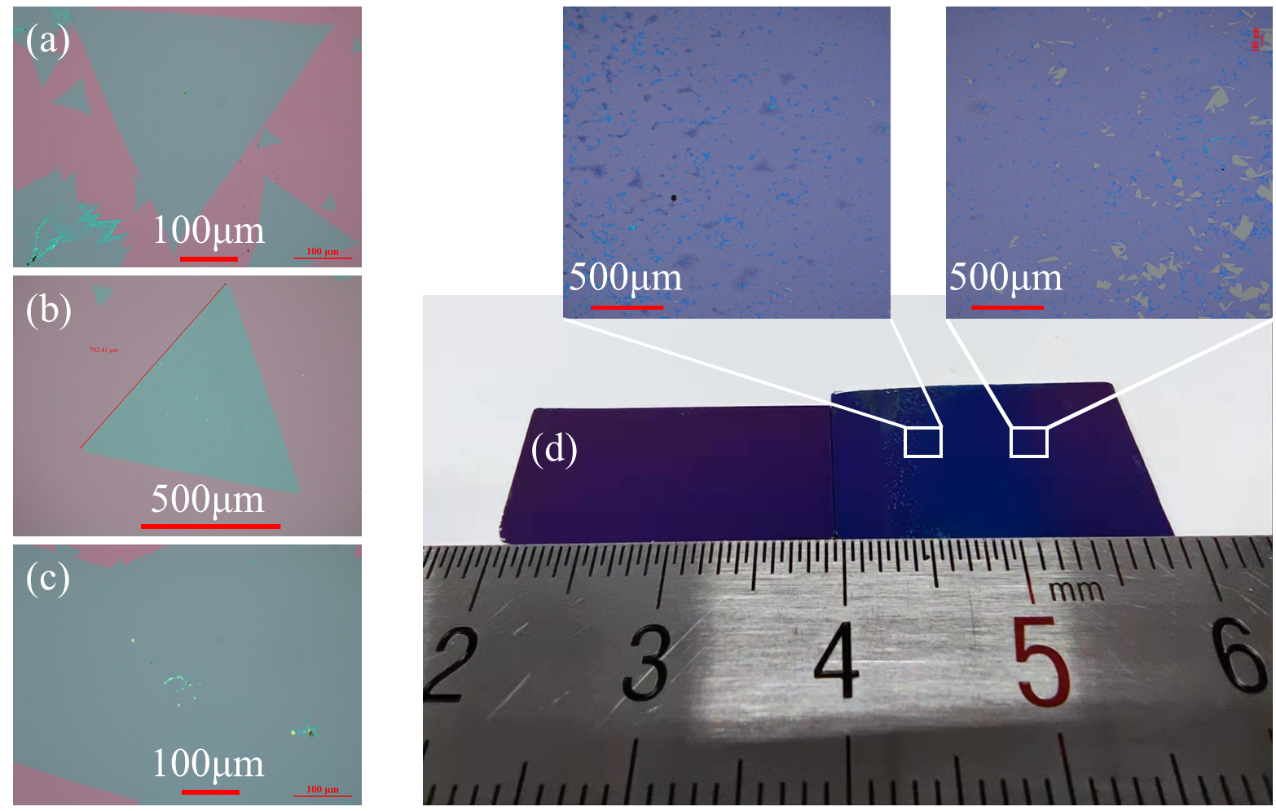


Figure S1. WS_2_ membranes synthesized by CVD method with different NaCl dosages. (a) 2 mg and (b) 4 mg and (c) 6 mg of NaCl powders are added and mixed sufficiently into the tungsten source. (d) When 8 mg of NaCl is added into the tungsten source and mixed sufficiently, centimeter scale of WS_2_ membranes are synthesized with obvious grains on the surfaces.

**S2. Details of the First principle calculations**

First-principles calculations based on density functions theory (DFT) are performed using QUANTUM ESPRESSO package.^[3-4]^ The Kohn-Sham orbital is expanded in a plane-wave basis with a cutoff energy of 80 Ry. The exchange correlation energy is described by generalized gradient approximation (GGA) scheme proposed by Perdew, Burke, and Ernzerhof (PBE) functional.^[5]^ A doping model of W atom in monolayer WS_2_ substitutionally doped by Er atom is constructed. The structure is optimized by the Broyden Fletcher Goldfarb Shanno (BFGS) method.^[6]^ WS_2_ has a hexagonal structure with the lattice constants of a = b = 3.191 Å, and C = 14.202 Å. Their bond angles are α=β=90°, γ=120°, which belongs to the P6_3_/mmc space group. The projector augmented plane wave (PAW) method is used to describe the interaction between electrons and ions. In order to facilitate the subsequent doping of Er atoms and improve the accuracy of calculation, a 20 Å vacuum layer is used to the intrinsic monolayer WS_2_ cell in the direction of C basic vector to eliminate the interference of periodic interlayer van der Waals (vdW) force, and expand the cell along the direction of a and b basic vector to obtain 2×2×1, 3×3×1, 4×4×1, and 5×5×1 supercells. On this basis, different Er doping concentrations can be obtained by replacing W atoms with Er atoms in the WS_2_ matrixes. For convergence criterion, the total energy and the Hellmann-Feynman force on each atom are set to be smaller than 1.0×10^-5^eV and 1.0×10^-4^eV/Å, respectively. Tkatchenko-Scheffler Vdw correction is applied to better take into account the interlayer Vdw force.^[7]^ The Brillouin-zone (BZ) is sampled using the Monkhorst-Pack method with different sizes of meshes for K point. For example, 3×3×1 of meshes for k-point is used for the 3×3×1 doping supercell.^[8]^ The number of k points are increased in the subsequent self-consistent calculations to ensure the accuracy for the electronic structure and optical properties of the monolayer WS_2_ doped system.

When constructing supercells, it is obvious that the side length of cell in real space will be expanded, and the cell vector will be reduced in its inverted space, which will lead to the expansion of the number of energy bands. This is not conducive to the analysis of results. The concentration of intrinsic defects and impurities in real materials is usually very low compared to the intrinsic matrix, supercell model is therefore used to study the properties of defects. However, the band structure of supercells cannot be directly compared with that of the original structure. To solve this problem, here, the energy band structures of supercells are transformed to the Brillouin region of protocells by using the effective band structure (EBS) theory, so that it is possible to compare the energy band structure of supercells containing defects with that of perfect protocells. The real energy band structure diagram cannot be directly reflected by the EBS data calculated by VASP, since the weight of each point in the energy band is involved in the data. To solve this problem, bubble diagram with weight (which representing the possibilities of electronic states in energy levels) is used to draw the energy bands of the S-vacancy, W-vacancy and Er-doped WS_2_ supercells.

**S2.1. 4×4×1 supercell for S-vacancy, W-vacancy, and Er filled in W-vacancy conditions**

4×4 supercell of S- and W- vacancy and Er-doped WS_2_ matrixes is calculated by the method as mentioned above. **Figure S2a** shows the schematic images of the S vacancy, W vacancy and Er-filled in W vacancy of supercell WS_2_, respectively. Figure S2b shows the energy band structures corresponding to Figure S2a. Similar to the condition in Figure 4b, weighted red bubble charts are used to represent the energy bands of the corresponding S-vacancy, W-vacancy and Er-doped WS_2_ monolayers, while the black curves represent the energy bands of the protocell WS_2_ for comparison. The energy bands of S-vacancy WS_2_ monolayer shows slight change compared with the protocell WS_2_ and 3×3×1 supercell WS_2_. Two deep defect energy levels with low weight in the forbidden band can be observed, which traps transition electrons in the forbidden band and thus lead to the reduction of the photoluminescent efficiency. In the meantime, high weight of valance band maximum exist in the high symmetric K point in Brillouin zone, indicating a relatively small impact of the S-vacancy on the direct transition. Compared with S-vacancy WS_2_, the energy bands change to more complicated in the W-vacancy WS_2_ supercell. Multiple trapping levels extend to the center of the forbidden band with uniform of low weight, and the valance band maximum (VBM) shows a small weight in the K point, indicating a very low possibility of carrier exist in the K point, and thus lower possibility of direct transition occur. Compared with the 3×3×1 supercell, the 4×4×1 supercell of W-vacancy WS_2_ shows a little higher of weight in the VBM at K point. However, it is also noted that W-vacancy in WS_2_ demonstrates more serious affection on the photoluminescent efficiency compared with S-vacancy as we calculated for the 3×3×1 supercell. For the third condition, when the W-vacancy is filled by Er-ion, both the VBM and conduction band minimum (CBM) moves upward in the K point with high weight, indicating high direct transition possibility, and thus the strongly enhanced of PL efficiency with a small redshift. To compare these three conditions, S-vacancy in the WS_2_ monolayer lead to relatively small impact on the PL intensity and small peak shift. W-vacancy deeply degenerates the PL efficiency. When Er filled in W-vacancy, the PL intensity is enhanced greatly with a redshifted peak. The result is consistent with that of the 3×3×1 supercell and well interprets the experimental result. Figure S2c shows the PDOS of the S-vacancy, W-vacancy, and Er-filled WS_2_ monolayer, respectively. Figure S2d-2i show the dependences of absorption index, refractive index, extinction index, reflectivity, energy loss, and dielectric constant, respectively, on the incident photon energy of the pristine WS_2_ and WS_2_(Er) with 4×4×1 supercell. The WS_2_(Er) sample also shows much higher of absortion, extinction, energy loss and imaginary part of dielectric indexes in the infrared region, which is similar to the result of 3×3×1 supercell. Amplitude exchanges are observed in the refractive, reflective, and real part of dielectric indexes compared with protocell WS_2_ monolayer.

^
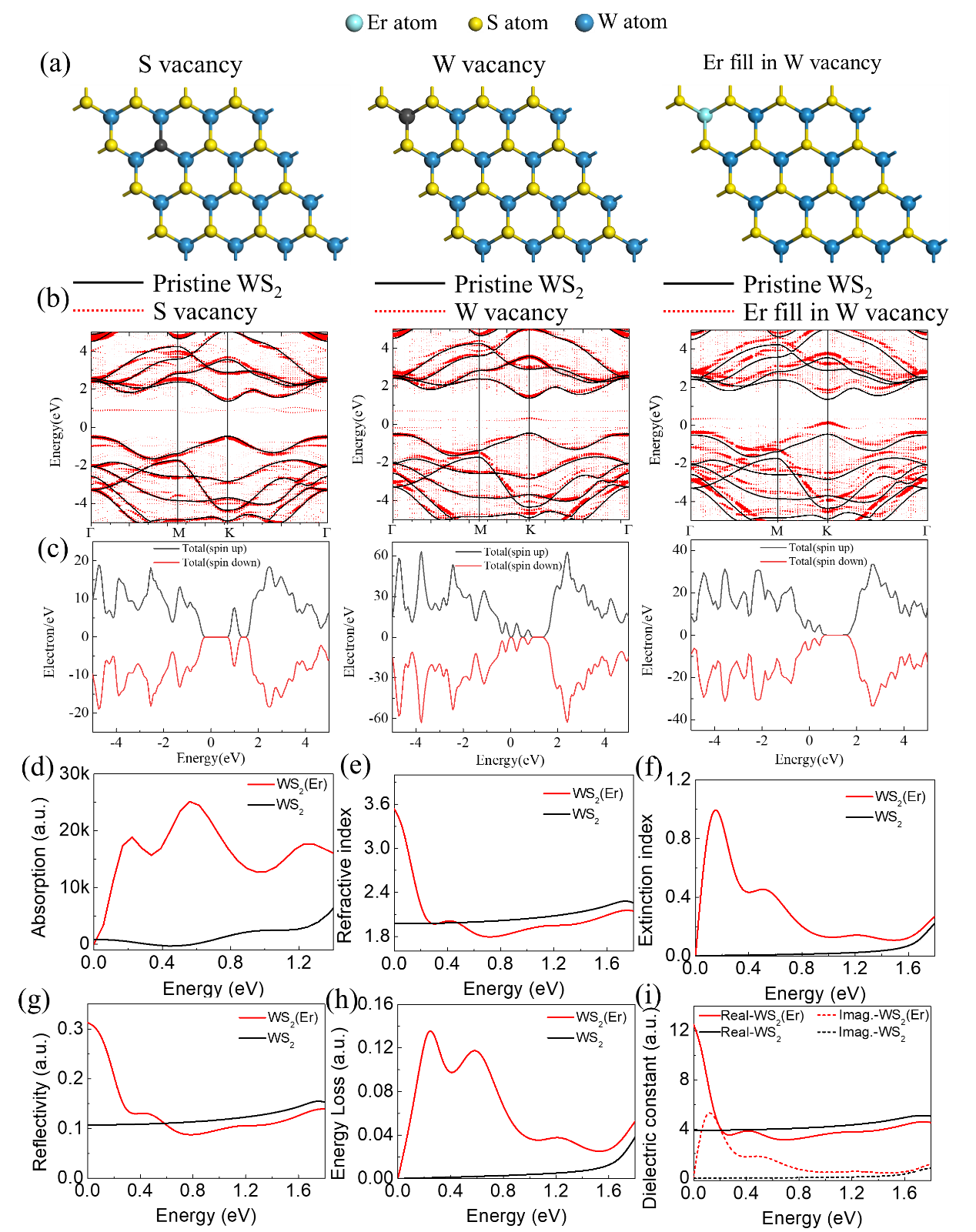
^

Figure S2. First principle calculations on the S-vacancy, W-vacancy, and W vacancy filled by Er of a 4×4×1 WS_2_ supercell. (a) Schematic illustration of the S-vacancy, W-vacancy, and W vacancy filled by Er of WS_2_ matrix. (b) Energy band structures corresponding to the vacancy conditions of WS_2_ supercell described in (a). Weighted bubble chart is used and displayed by the red dotted lines representing the band structures of vacancy and Er doped WS_2_ monolayers, while the black solid curves represent band structure of the pristine monolayer WS_2_. (c) PDOS of monolayer WS_2_ under the S-vacancy, W-vacancy, and Er-filled in W vacancy conditions, respectively. (d)-(i) Dependences of absorption index, refractive index, extinction index, reflectivity, engergy loss, and dielectric constant respectively, on the incident photon energy (from 0-1.8eV) of the pristine WS_2_ and WS_2_(Er) monolayer.

**S2.2. 5×5×1 supercell for S-vacancy, W-vacancy, and Er filled in W-vacancy conditions**

5×5×1 supercell for S-vacancy, W-vacancy, and Er filled in W-vacancy of WS_2_ monolayer are calculated. **Figure S3a** shows the energy bands of the supercell under S-vacancy, W-vacancy and Er-filled in W-vacancy condtions. It is obvious that when the supercell expands, the defect energy levels are reduced and their weight in the bobble charts also decreased, indicating palliated trapping effect caused by the vacancy compared with the energy bands of 3×3×1 and 4×4×1 supercells of the vacancy conditions. In particular, for the S-vacancy monolayer, the CBM shows a little downward offset. For the W-vacancy monolayer, the CBM and VBM energy levels in the K point almost overlapped with those of the protocell WS_2_ monolayer, except three trapping levels exist in the forbidden band, indicating the unchanged shape of photoluminescence with reduced quantum efficiency. This is well consistent with the experimental result. For the Er-doped WS_2_ sample, the VBM is enhanced in the K point, but its weight also increased significantly, corresponding to a redshift of the photoluminescence with promoted quantum efficiency, which is also well consistent to the experimental result. Figure S3b shows the PDOS of the three conditions corresponding to (a).


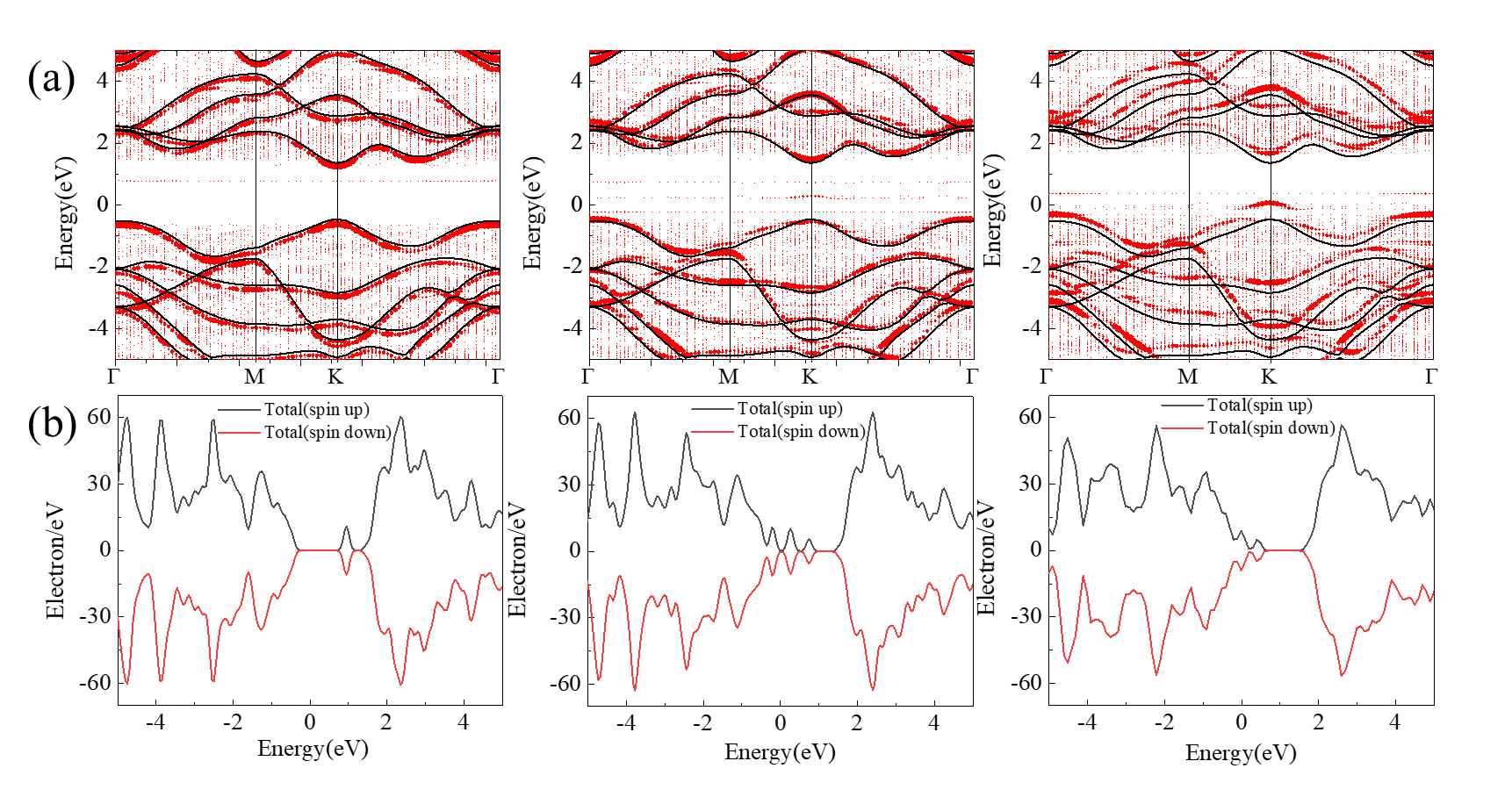


Figure S3. First principle calculations on the S-vacancy, W-vacancy, and W vacancy filled by Er of a 5×5×1 WS_2_ supercell. (a) Schematic illustration of the S-vacancy, W-vacancy, and W vacancy filled by Er of WS_2_ matrix. (b) Energy band structures corresponding to the vacancy conditions of WS_2_ supercell described in (a). Weighted bubble chart is used as the 3×3×1 and 4×4×1 supercell.

**S3. Dark current of photodetectors based on WS_2_ and WS_2_(Er) monolayers**

Dark current (*I-V*) of photodetectors based on WS_2_ and WS_2_(Er) monolayers are measured on a shielded probe station connected to a Keithley semiconductor analyzer (SCS4200) by a standard DC technique, as shown in **Figure S4**. Both of the two type of devices show linear I-V curve passing through the origin, which indicates Ohmic contacts between the electrodes and the WS_2_ and WS_2_(Er) membranes. When 1 V of Vds is applied on the devices in dark, the WS_2_ device shows a channel current of 0.65 nA, while the WS_2_(Er) device shows a channel current of 7.5 nA. This means 11 times of dark current are produced under 1 V of V_ds_ in the WS_2_(Er) device compared with the WS_2_ device. Because similar shape, size, and thickness of WS_2_ and WS_2_(Er) monolayers are selected during the preparation of devices, and they are also fabricated in the same course of processing, the enhanced dark current from WS_2_(Er) device is probably originates from the high gain that produced by the high concentration of Er ion doping.

Figure S4 Dark current (*I-V*) of photodetectors based on WS_2_ and WS_2_(Er) monolayers.

**S4. Comparison of the photoresponsive characteristics between WS_2_(Er) photodetector and photodetector based on other 2D materials**

To assess the photoresponse performance of the present Er-doped WS_2_, we summarize in Table 1 the reported photoresponse properties in literatures for other semiconducting vdW materials together with their experimental conditions for horizontal contrast. The present photoresponse properties of the WS_2_(Er) device are especially marked for its fast photoresponse speed, high external quantum efficiency and broad photoresponse bandwidth from ultraviolet to infrared regions compared with other semiconducting vdW materials with compatable device structures, which make the monolayer WS_2_(Er) especially promising for very fast and broadband photodetection applications with high gain. This provides a robust and essential building block for n-channel transistors, complementary circuits, and tunneling field-effect transistors. What’s more, the successful fabrication of centimeter scale of WS_2_(Er) membrane indicate the great potential of the technique and material to be used in photoelectronics and electronics applications.

Table 1. Comparison of the Critical Parameters for the Reported vdW-Material based Photodetectors.

| **Material** | **Structure and dielectric** | **Measuring conditions** | **R_λ_**  **(mA/W)** | **τ_rise_**  **(ms)** | **Response range (nm)** | **EQE**  **(%)** | **Ref.** |
| --- | --- | --- | --- | --- | --- | --- | --- |
| WS_2_(Er)  ～1 nm  (CVD) | Photodetector on SiO_2_/Si | V_ds_ = 1 V, λ = 635 nm, P_d_ = 180mW/cm^2^ | 994.5 | 0.19 (rise) | ultraviolet to infrared | 194.6 | This work |
| WS_2_  ～1 nm  (CVD) | Photodetector on SiO_2_/Si | V_ds_ = 1 V, λ = 635 nm, P_d_ = 180mW/cm^2^ | 7.8 | 25  (rise) | Ultraviolet to visible | 1.5 | This work |
| WS_2_  ～1 nm  (CVD) | 0.79 nm on SiO_2_/Si | V_ds_=9V,  λ = 635 nm, P_d_=1039mW/cm^2^ | 3.7 | 42 | ND | ND | [9] |
| Monocrystal Si | Silicon solar cell | ND | ND | ND | 350-1100 nm | 95 | [10] |
| 0.8nm-thick  MoS_2_ | FET, back-gate,  300nm SiO_2_ | V_ds_ = 3 V, V_g_ = 70 V, λ = 532nm, | 8 | 50 (rise) | Visible to 800nm | ND | [11] |
| 8nm-thick black phospherene | FET, back-gate, 285nm SiO_2_ | V_ds_ = 0.1 V, V_g_ = 0V, λ = 640 nm, P_d_ = 10 pW | 4.8 | 1 (rise) | Visible to 997nm | ND | [12] |
| 30nm-thick MoS_2_ | FET, top-gate, 70nm Al_2_O_3_ | V_ds_ = 0.2 V, V_g_ = 70 V, λ = 532nm, | ＞100 | ＞1000 | Visible to 850nm | ND | [13] |
| 6nm-thick WS_2_ | 2-terminal, on quartz | V_ds_ = 30 V, λ = 458 nm, P =2 mW | 0.02 | 5.3 | Visible | ND | [14] |
| Trilayer WSe_2_ | ND | V_ds_ = 2 V, λ = 532 nm | 100 | 0.04 | 590-740nm | ND | [15] |
| 4.23nm-thick GaSe | 2-terminal, ND for SiO_2_ thickness | V_g_ = 0 V, λ = 254 nm, P = 1 mW/cm^2^ | 2800 | 20 | UV to 700nm | ND | [16] |
| p-GaTe/n-MoS_2_ | Vdw heterojunction | λ=633 nm, V_DS_=0 V | 1.365 | ND | ND | 266 | [17] |
| p-GaSe/n-InSe | Vdw heterojunction | λ=410 nm | 0.021 | ND | ND | 9.3 | [18] |
| p-GeSe/n-MoS_2_ | Vdw heterojunction | λ=532 nm | 0.105 | ND | ND | 24.2 | [19] |
| 60nm to 230nm-thick GeSe (indirect bandgap) | FET, back-gate, 300nm SiO_2_ | V_ds_ = 1 V, V_g_ = 0V, λ = 808 nm, P_d_ = 283mW/cm^2^ | 3500 | 100 (rise) | Visible to infrared | ND | [20-22] |

**References:**

[1] M. Kim, J. Seo, J. Kim, J. S. Moon, J. Lee, J. H. Kim, J. Kang, H. Park, ACS Nano, 15, 3038, 2021.

[2] S. Li, S. Wang, D.-M. Tang, W. Zhao, H. Xu, L. Chu, Y. Bando, D. Golberg, G. Eda, Appl. Mate. Today, 1, 60, 2015.

[3] W. Kohn, L. J. Sham, Phys. Rev. 140, A1133, 1965.

[4] P. Giannozzi, S. Baroni, N. Bonini, M. Calandra, R. Car, C. Cavazzoni, D. Ceresoli, G. L. Chiarotti, M. Cococcioni, I. Dabo, A. Dal Corso, S. de Gironcoli, S. Fabris, G. Fratesi, R. Gebauer, U. Gerstmann, C. Gougoussis, A. Kokalj, M. Lazzeri, L. Martin-Samos, N. Marzari, F. Mauri, R. Mazzarello, S. Paolini, A. Pasquarello, L. Paulatto, C. Sbraccia, S. Scandolo, G. Sclauzero, A. P. Seitsonen, A. Smogunov, P. Umari, R. M. Wentzcovitch, J. Phys. Condens. Matter. 21, 395502, 2009.

[5] J. P. Perdew, K. Burke, M. Ernzerhof, Phys. Rev. Lett. 77, 3865, 1996.

[6] T. H. Fischer, J. Almlof, J. Phys. Chem. 96, 9768, 1992.

[7] A. K. Singh, R. G. Hennig, Appl. Phys. Lett. 105, 042103, 2014.

[8] H. J. Monkhorst, J. D. Pack, Phys. Rev. B, 13, 5188, 1976.

[9] J. Shen, J. Yan, L. Zhan, C. Wu, B. Ge, X. Wang, H. Wang, Q. Cui, D. Yang, H. Zhang, Nanotechnology, 32, 505603, 2021.

[10] S. Chander, A. Purohit, A. Nehra, S. Nehra, M. Dhaka, Int. J. Renewable Energy Res. 5, 41, 2015.

[11] Z. Yin, H. Li, H. Li, L. Jiang, Y. Shi, Y. Sun, G. Lu, Q. Zhang, X. Chen, H. Zhang, ACS Nano, 6, 74, 2012.

[12] M. Buscema, D. J. Groenendijk, S. I. Blanter, G. A. Steele, H. S. van der Zant, A. Castellanos-Gomez, Nano Lett. 14, 3347, 2014.

[13] W. Choi, M. Y. Cho, A. Konar, J. H. Lee, G. B. Cha, S. C. Hong, S. Kim, J. Kim, D. Jena, J. Joo, S. Kim, Adv. Mater. 24, 5832, 2012.

[14] N. Perea-López, A. L. Elías, A. Berkdemir, A. Castro-Beltran, H. R. Gutiérrez, S. Feng, R. Lv, T. Hayashi, F. López-Urías, S. Ghosh, B. Muchharla, S. Talapatra, H. Terrones, M. Terrones, Adv. Funct. Mater. 23, 5511, 2013.

[15] N. R. Pradhan, J. Ludwig, Z. Lu, D. Rhodes, M. M. Bishop, K. Thirunavukkuarasu, S. A. McGill, D. Smirnov, L. Balicas, ACS Appl. Mater. Interfaces, 7, 12080, 2015.

[16] P. Hu, L. Wang, M. Yoon, J. Zhang, W. Feng, X. Wang, Z. Wen, J. C. Idrobo, Y. Miyamoto, D. B. Geohegan, K. Xiao, Nano Lett. 13, 1649, 2013.

[17] S. Yang, C. Wang, C. Ataca, Y. Li, H. Chen, H. Cai, A. Suslu, J. C. Grossman, C. Jiang, Q. Liu, S. Tongay, ACS. Appl. Mater. Interfaces, 8, 2533, 2016.

[18] F. Yan, L. Zhao, A. Patane, P. Hu, X. Wei, W. Luo, D. Zhang, Q. Lv, Q. Feng, C. Shen, K. Chang, L. Eaves, K. Wang, Nanotechnology, 28, 27LT01, 2017.

[19] Y. Xin, X. Wang, Z. Chen, D. Weller, Y. Wang, L. Shi, X. Ma, C. Ding, W. Li, S. Guo, R. Liu, ACS Appl. Mater. Interfaces, 12, 15406, 2020.

[20] H. Zhao, Y. Mao, X. Mao, X. Shi, C. Xu, C. Wang, S. Zhang, D. Zhou, Adv. Funct. Mater. 28, 1704855, 2017.

[21] W. C. Yap, Z. Yang, M. Mehboudi, J.-A. Yan, S. Barraza-Lopez, W. Zhu, Nano Res. 11, 420, 2017.

[22] B. Mukherjee, Y. Cai, H. R. Tan, Y. P. Feng, E. S. Tok, C. H. Sow, ACS Appl. Mater. Interfaces, 5, 9594, 2013.
